# Dental Disease an Etiological Factor in Affections of the Eye

**Published:** 1902-01-15

**Authors:** Juler Cundell

**Affiliations:** Cincinnati, O.


### DENTAL DISEASE AN ETIOLOGICAL FACTOR IN AFFECTIONS OF THE EYE.

BY CUNDELL, JULER M.D., CINCINNATI, O.

In studying the fifth pair of nerves with its peculiar reflexes and its intimate association with certain other cranial nerves we can understand how irritation in one of its trunks may cause trouble in some distant structure supplied by a branch of the same nerve, or along the course of another cranial nerve with which the fifth is associated. The eye being a very sensitive and delicate structure, is very apt to become the seat of reflex irritation, and such irritation may spread rapidly, involving one structure of the eye after another until the organ becomes destroyed.

In affections of the eye where the cause is not readily detected, the teeth should be thoroughly examined, as an exposed pulp in some lurking position if discovered and treated early may avoid the most disasterous result in the eye. Travers and Frick in 1824 and 1826 were the first to make any reference to this subject when they spoke of difficult dentition as a cause of strabismus.

Reflex sympathetic ophthalmia had long been known where inflammation of one eye was followed after variable duration by inflammation of the uninjured eye.

It was known also that injuries affecting the first (ophthalmic) division of the fifth pair might affect the eye of that side; and it was thought that damage to other branches of the fifth pair might be the cause of ophthalmic troubles.

Since that time the fact that dental irritation may under certain circumstances set up reflex irritation in the eye has been abundantly proven.

A case was mentioned by Gayet in 1886, in which disturbance in the eye was produced by a tooth fixed on a pivot, the symptoms appeared and disappeared accordingly as the tooth was removed or replaced,

In asthenopia without any apparent cause, the teeth should always be examined, Cases are recorded in which disturbance in vision had been produced, and after the lapse of several months the offending tooth had been extracted followed by complete restoration of sight.

Abscess of the cornea after resisting treatment has been relieved by the extraction of carious teeth. M. Fieuzal observed so many of these cases of co-relation between ocular and dental affections, that he had urged that a dental clinic should be annexed to the Ouinze Vingt's hospital for the blind.

An army surgeon having placed a man under chloroform who suffered intense and persistent pain in one eye which had resisted ordinary treatment; removed the wrong eye, the man recovering from the anesthetic had the pain as bad as ever; when it was discovered that the pain was due to an abscess in the root of a tooth which being removed cured the eye. Erroneous diagnosis have been made by other surgeons, and iridectomies have been performed, the cause later being detected in the teeth.

